# Exosomal miR-320d promotes angiogenesis and colorectal cancer metastasis via targeting GNAI1 to affect the JAK2/STAT3 signaling pathway

**DOI:** 10.1038/s41419-024-07297-y

**Published:** 2024-12-18

**Authors:** Yawen Wu, Jie Zhang, Guanghao Li, Li Wang, Yajing Zhao, Baibing Zheng, Fanfeng Lin, Li Xie

**Affiliations:** 1 Shandong Provincial Key Laboratory of Precision Oncology, Shandong Cancer Hospital and Institute, Shandong First Medical University and Shandong Academy of Medical Sciences, Jinan, Shandong China; 2 Clinical Laboratory, Children’s Hospital Affiliated to Shandong University, Jinan, Shandong Province China; 3 Department of Clinical Laboratory, Hangzhou Fuyang District First People’s Hospital, Hangzhou, Zhejiang China; 4 Department of Clinical Laboratory, Shandong Cancer Hospital and Institute, Shandong First Medical University and Shandong Academy of Medical Sciences, Jinan, Shandong PR China; 5 Department of Laboratory, Tianjin Medical University Cancer Institute and Hospital, Tianjin’s Clinical Research Center for Cancer, Key Laboratory of Cancer Prevention and Therapy, National Clinical Research Center for Cancer, Tianjin, China

**Keywords:** Metastasis, Tumour angiogenesis

## Abstract

Colorectal cancer is a common malignant tumor, whose growth and metastasis are influenced by numerous factors. MicroRNAs have garnered increasing attention in recent years due to their involvement in tumor development. Exosomes are involved in intercellular signaling and influence tumor development by promoting tumor cell proliferation and metastasis through activation of angiogenesis and other mechanisms. This study aimed to investigate how the exosomes containing miR-320d from colorectal cancer (CRC) cells promote colorectal cancer metastasis by regulating angiogenesis. CRC-derived exosomes containing miR-320d can be transferred to vascular endothelial cells, facilitating their proliferation, invasion, migration, and angiogenesis. By targeting GNAI1, miR-320d in these exosomes reduces GNAI1 levels in endothelial cells, causing more JAK2/STAT3 activation and VEGFA production. This ultimately enhances the migration and angiogenic capacity of vascular endothelial cells. Moreover, CRC patients with high levels of miR-320d in their blood respond better to treatment with bevacizumab. In vivo experiments further proved the role of miR-320d from CRC exosomes in increasing tumor size, blood vessel formation, and the spread of cancer to the liver. In this study, we have demonstrated that exosomal miR-320d promotes cancer cell metastasis and enhances angiogenesis by downregulating GNAI1 expression and enhancing JAK2/STAT3.

## Introduction

According to the latest global cancer statistics, colorectal cancer ranks second in terms of death rate, and over half of patients with this illness develop liver metastases. Furthermore, liver metastases from colorectal cancer are the main cause of death in these patients [1]. Angiogenesis plays a critical role in tumor metastasis [2, 3]. Angiogenesis is the natural process through which new blood vessels are formed from existing capillaries or small veins, involving the growth, sprouting, movement, and development of the inner lining in blood vessel cells [4]. Previous studies have shown that solid tumors have more blood vessels inside them compared to the surrounding tissue, providing oxygen and nutrients to the tumor tissue, which helps in its growth and spread [5]. Several drugs that inhibit angiogenesis, such as bevacizumab and rituximab, have been approved by the FDA. These drugs can block the formation of new blood vessels, thus reducing blood supply to tumors and slowing down tumor growth and spread.

Exosomes are tiny vesicles ranging from 30 nm to 150 nm in diameter, carrying various complex RNAs and proteins. They play a crucial regulatory role by transporting these molecules for cell-to-cell communication and information exchange [6]. Many studies have highlighted the essential biological functions of exosomes in different physiological and pathological processes. They engage in intercellular signaling and regulate cell growth, proliferation, and differentiation. In cancer development, exosomes might contribute to tumor cell proliferation and metastasis by activating angiogenesis and modulating immune responses, thus impacting tumor progression [7, 8].

MicroRNAs (miRNAs) are short, non-coding RNA molecules, typically around 22 nucleotides in length, encoded by endogenous genes. They play a critical role in post-transcriptional gene expression regulation in both plants and animals [9]. An increasing body of evidence suggests the significant involvement of exosomal miRNAs in cancer. Once released and taken up by nearby or distant cells, these enclosed miRNAs facilitate tumor growth, invasion, metastasis, and angiogenesis by regulating cancer cell proliferation and migration [10]. In a previous study conducted in our laboratory, it was observed that the expression of serum exosomal miR-320d was significantly elevated in patients with metastatic colorectal cancer [11]. Therefore, this study focuses on elucidating the mechanism of exosomal miR-320d in the metastasis of colorectal cancer.

## Materials and methods

### Cell culture

Human vascular endothelial cells (HUVEC, EA.hy926) and colorectal cancer cells SW480 and HCT-116 were obtained from the Cell Bank of the Chinese Academy of Sciences (Shanghai, China). HUVEC and EA.hy926 cells were cultured in DEME medium (Gibco, Carlsbad, CA, USA), while SW480 and HCT-116 cells were cultured in 1640 medium (Gibco, Carlsbad, CA, USA) without exosomes. The medium was supplemented with 10% FBS (Gibco) and 1% antibiotics (penicillin/streptomycin at a concentration of 100 U/ml). All cells were maintained at 37 °C with 5% CO_2_. Colorectal cancer cells were transfected with miR-320d mimic/inhibitor (Ribobio China) using Lipofectamine 3000 reagent (Invitrogen) following the manufacturer’s instructions. Exosomes produced by these cells were collected and used to incubate vascular endothelial cells. HUVEC and EA.hy926 cell lines were identified by STR analysis.

### Collection and extraction of exosomes

Complete medium from SW480 and HCT-116 cells was collected and underwent sequential centrifugation steps: first at 300 × *g* for 10 min (4 °C) to remove cells and dead cells, then at 2000 × *g* for 10 min (4 °C) to eliminate cellular debris, followed by 10,000 × *g* for 30 min (4 °C) to remove macrovesicles, and finally at 100,000 × *g* for 2 h (4 °C) to isolate supernatant exosomes. Similarly, serum from CRC patients and healthy volunteers underwent sequential centrifugation steps: first at 3000×*g* for 10 min (4 °C), followed by 10,000 × *g* for 30 min (4 °C), and finally at 100,000 × *g* for 2 h (4 °C) to obtain exosomes.

### Transmission electron microscopy (TEM) assay

Exosomes extracted from SW480 and HCT-116 cells were subjected to TEM analysis as follows: 15 μl of purified exosomes were placed on a copper grid for 1 min, followed by the addition of 50 μl of 1% glutaraldehyde for 5 min. Subsequently, 100 μl of distilled water was added to the copper grid and left for 2 min, then 15 μl of 2% uranyl acetate staining solution was added and stained for 5 min at room temperature. The exosomes on the copper mesh were dried, and their morphology was observed using transmission electron microscopy, and the images were saved.

### qNano

The exosome samples obtained through ultracentrifugation were resuspended in PBS and placed on ice. The qNano instrument (Izon Sciences Ltd) was then activated to measure the particle size of the exosomes. Subsequently, the data were analyzed using Izon Control Suite v.3.3.2.2001 software, and corresponding images were generated.

### Exosome uptake assay

Exosomes extracted via ultracentrifugation were initially labeled with PKH-67 fluorescent dye and then suspended in exosome-free DMEM medium for endothelial cell culture (HUVEC and EA.hy926). Endothelial cell samples were collected and subjected to immunofluorescence analysis at 0, 6, and 12 h. Fluorescent images of the cells were captured using confocal laser microscopy to assess exosome uptake.

### In vitro proliferation, migration and wound-healing assays

Cell Proliferation Assay: Endothelial cells, post-exosome incubation or transfection, were counted and seeded at a density of 1000 cells per well in 96-well plates. The plates were then incubated at 37 °C with 5% CO_2_. Subsequently, the cells were treated with CCK8 reagent for 1 h daily at a consistent time, and the absorbance at 450 nm was determined using an enzyme marker.

Cell Migration Assay: Endothelial cells, counted after exosome incubation or transfection, were seeded into the upper chamber of 0.8μm Transwell and incubated for 8 h at 37 °C with 5% CO_2_. After incubation, the migrated cells were fixed with formaldehyde for 30 min, stained with 0.1% crystal violet solution for another 30 min, and then counted and photographed using a light microscope. Subsequent analysis was conducted using Image J.

Cell Wound-Healing Assay: Endothelial cells, counted after exosome incubation or transfection, were seeded in six-well plates at a density of 1.5 × 10^5^ cells per well. Once the cells reached adequate confluence, artificial wounds were created by scratching with a 200 μl sterile yellow lance tip. Photographs were taken at 0 h and 24 h, and subsequently analyzed and counted using Image J.

### Angiogenesis experiment

A volume of 250 μl of matrix gel (Corning, 356234) was dispensed into pre-cooled 24-well plates and allowed to solidify. Subsequently, endothelial cells subjected to exosome incubation or transfection were counted and seeded into the wells. Following an incubation period of 6 to 8 h at 37 °C with 5% CO_2_, photographs were captured and subjected to statistical analysis.

### Data acquisition and processing

We utilized TCGA (https://www.cancer.gov/ccg/research/genome-sequencing/tcga) and GSE datasets (GSE48267; GSE110402; GSE98406; GSE39833; GSE81582) to extensively investigate the expression levels of miR-320d and GNAI1 in tissues and serum exosomes of colorectal cancer patients and healthy volunteers. We evaluated the angiogenic capacity scores of colorectal tissue and normal tissue of the TCGA cohort using the single-sample gene set enrichment analysis (ssGSEA) method with the angiogenic gene set available on the official GSEA website (http://www.gsea-msigdb.org/). Additionally, we analyzed the impact of high or low angiogenic capacity scores on the survival of patients with colorectal cancer.

### Collection of clinical specimens

The researchers collected serum samples from 70 healthy volunteers and 108 patients diagnosed with colorectal cancer (CRC), as well as 38 pairs of colorectal cancer tissues and corresponding paracancerous tissues, which were stored at −80 °C at the Affiliated Cancer Hospital of Shandong First Medical University. Requirements for inclusion in the health examination specimen include the absence of benign and malignant tumors, nodules, colon polyps, and any other endocrine, immune, or metabolic disorders. CRC patients were diagnosed with colorectal cancer by a combination of clinical, pathological, and radiological diagnostic methods, and tumor stage was determined according to the American Joint Committee on Cancer (AJCC) 8th edition, and the requirements for enrollment were the absence of a primary tumor elsewhere, the absence of anti-tumor therapy, and the absence of other endocrine, immune, or metabolic disorders. The study protocol was approved by the Ethics Committee of the Affiliated Cancer Hospital of Shandong First Medical University and was conducted in accordance with the principles of the Declaration of Helsinki.

### RNA extraction, reverse transcription and q-PCR

Total RNA was extracted from tissue, cellular, and exosomal samples using Trizol. Subsequently, reverse transcription into cDNA was performed using miRNA or mRNA reverse transcription kits. The samples underwent 40 cycles of real-time quantitative PCR (qPCR) using the Roche LightCycler 480 system to detect both the target and internal reference genes. miR-320d forward primer (GAAAAGCTGGGTTGAGAGGAAA); GNAI1 primer (Forward: ATGCACGCCAACTCTTTGTG; Reverse: GCTGGTACTCTCGGGATCTG); VEGFA primer (Forward: ACTGCCATCCAATCGAGACC; Reverse: CTGGCCTTGGTGAGGTTTGA). To assess fold expression, the 2^−^^ΔΔCt^ method was employed, and relative RNA expression levels were calculated using the formula ΔCT = CT^RNA^ − CT^U6^. U6 served as the endogenous control for miR-320d, while actin was used for GNAI1 and VEGFA.

### Western blots

Cells or exosomes were lysed with cell lysis buffer (beyotime, China), and the total cellular protein concentration was determined using the BCA Protein Assay Kit (ThermoFisher), supplemented with 0.5 mM PMSF (beyotime, China) and a phosphatase inhibitor (beyotime, China). Subsequently, 30 μg of protein was separated by SDS-PAGE and transferred to a PVDF membrane. The membranes were then blocked with TBST containing 5% skimmed milk for 1–2 h at room temperature and subsequently incubated overnight at 4 °C with primary antibodies: anti-human GNAI1 (1:1000 proteintech), VEGFA (1:500 abcam), P-STAT3 (1:500 CST), STAT3 (1:500 CST), p-JAK2 (1:500 CST), JAK2 (1:500 CST), GAPDH (1:5000 proteintech); CD9 (1:500 CST), HSP60 (1:500 CST), GM130 (1:500 CST). The next day, the membranes were incubated with an HRP-coupled anti-rabbit antibody (1:5000 proteintech) for 1 h at room temperature. Protein bands were visualized using ECL blotting detection reagent (ThermoFisher, USA), followed by development and fixation with X-ray film.

### Animal experiment

For the tumor xenograft model, twelve 6-week-old male nude mice (BALB/c) were subcutaneously injected in the hind limbs with 5 × 10^6^ SW480 cells containing luciferase. Tumor formation was monitored regularly, and tumor volume, calculated in mm^3^ using the formula (length*width^2^)/2, was measured upon tumor development. The mice were randomly assigned to the mimics NC-Exos group, miR-320d mimics-Exos group, and received intra-tumoral injections of 10 μg exosomes every 4 days. Tumor volume was recorded, and bioluminescence was monitored using the IVIS Spectral In Vivo Imaging System (PerkinElmer, USA) after 24 days. Subsequently, the tumors were excised, photographed, weighed, fixed, and stained.

For the liver metastasis model, twelve 6-week-old male nude mice were divided into the mimics NC-Exos group and miR-320d mimics-Exos group. A total of 5 × 10^6^ SW480 cells pretreated with exosomes were injected via the tail vein. After 4 weeks, the livers of the mice were photographed, fixed, and stained. The mice were obtained from Spivey Bioscience.

For matrigel implant assay model, twelve 5- to 6-week-old Balb/c mice, we performed subcutaneous injection of a mixture of Matrigel (354234 Corning) and PBS containing exosomes into the abdominal cavity. One week later, the mice were executed, the skin was incised, the matrix plugs were removed, fixed in formalin overnight, paraffin-embedded, and sectioned. Staining with hematoxylin and eosin (H&E) was performed to observe angiogenesis within the gel. All procedures were approved by the Ethics Committee of the Affiliated Cancer Hospital of Shandong First Medical University, and the investigators were blinded to the group allocations during the experiment.

### Histopathological analysis

The mouse tumor tissues underwent submersion in formalin solution at room temperature for over 24 h, followed by dehydration, paraffin embedding, and subsequent sectioning. Anti-CD31 and Anti-VEGF treatment was applied to the tissues, followed by a secondary antibody, to assess immunoreactivity. Finally, the tissues were stained using DAB substrate (Thermo Fisher Scientific). Mouse liver tissues were fixed in 4% paraformaldehyde for over 24 h, then embedded in paraffin and sectioned. Hematoxylin and eosin (H&E) staining was performed, and the morphological changes in these tissues were examined using a light microscope with various fields of view.

### Screening of the target gene GNAI1 and dual-luciferase reporter assay

Initially, we obtained transcriptomic data (FPKM format) from the TCGA database for cohorts of colon (TCGA-COAD) and rectal (TCGA-READ) cancer patients and extracted the expression levels of miR-320d for each patient. Subsequently, we conducted a Spearman correlation analysis to explore mRNAs negatively correlated with miR-320d expression. mRNAs with a correlation coefficient *R* < 0 and *P* < 0.05 were identified as potential targets of miR-320d. We then used the miRDB database (https://mirdb.org/) and TargetScan database (https://www.targetscan.org/vert_80/) to predict downstream targets of miR-320d. Finally, to achieve more precise results, we performed an intersection analysis of the aforementioned results, identifying the final target gene GNAI1. In the TargetScan database, we determined the binding sites between miR-320d and the 3’UTR of GNAI1. The 3’UTR of GNAI1 was inserted into the psiCHECKTM-2 vector (Promega, Madison, WI, USA). Following co-transfection of the GNAI1 3’UTR construct and either miR-320d mimic or mimic NC in 293T, CRC, and HUVEC cells using Lipofectamine 3000 for 24 h, the cells were lysed, appropriate substrates were added, and fluorescent signals were detected for statistical analysis.

### Statistical analysis

All analyses were performed using SPSS 21.0 software (IBM, Ehningen, Germany) and GraphPad Prism version 8.0 software (San Diego, CA, USA), with a significance level set at *P* < 0.05. The Shapiro–Wilk test was used to assess the normality of the data distribution. For normally distributed data, unpaired *t*-tests were conducted, for non-normally distributed data, the Mann–Whitney test was applied. In paired data, paired *t*-tests were used for normally distributed variables, while non-normally distributed variables were analyzed using the Wilcoxon signed-rank test. Spearman correlation analysis was employed to evaluate the degree of linear relationship between miR-320d and GNAI1. The Kaplan–Meier method was utilized to analyze survival curves, with significance evaluated using the log-rank test.

## Results

### Colorectal cancer-derived exosomes are delivered to vascular endothelial cells

**Fig. 1:**
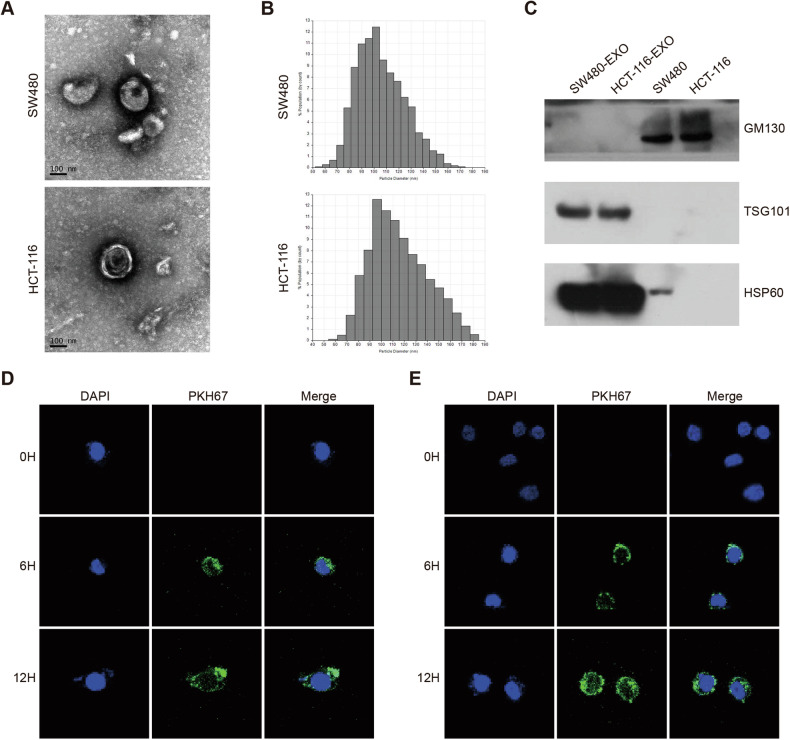
Morphological identification and exosome delivery. **A** TEM images illustrating exosomes isolated from SW480 and HCT-116 cell cultures. (Scale: 100 nm). **B** The qNano system was employed to detect exosomes isolated from SW480 and HCT-116 cell cultures, with diameters primarily ranging from 80–130 nm. **C** Western blot analysis of exosomal protein markers TSG101 and HSP60, along with the cellular protein marker GM130. **D**, **E** Internalization of exosomes by vascular endothelial cells HUVEC (**D**) and EA.hy926 cells (**E**) after 6 and 12 h of culture, visualized through PKH67 labeling of exosomes and DAPI labeling of vascular endothelial nuclei.

To assess the reliability of exosome extraction, we analyzed the precipitates obtained through ultracentrifugation from the culture medium of SW480 cells and HCT-116 cells, focusing on their morphology and size. The results revealed that the precipitates exhibited a characteristic rounded lipid bilayer membrane-like structure (Fig. 1A), consistent with the typical morphology of exosomes. Additionally, the vesicles obtained through ultracentrifugation displayed a diameter ranging from 50 nm to 180 nm (Fig. 1B), closely aligning with the expected particle size of exosomes. To further validate the exosomes’ properties, Western blot experiments were conducted to identify protein molecules specific to exosomes. Exosomes showed significant expression of exosome membrane structural proteins CD9 and HSP60, while GM130 protein was absent (Fig. 1C). These findings unequivocally support the credibility of the exosome extraction technique. To investigate the potential transport of exosomes released by CRC cells to vascular endothelial cells, we utilized PKH67 labeling to mark the exosomes. These labeled exosomes were then co-incubated with HUVEC and EA.hy926 cells for a specific duration. Subsequent observations using confocal microscopy demonstrated the delivery of CRC cell-released exosomes to both HUVEC cells (Fig. 1D) and EA.hy926 cells (Fig. 1E).

### Exosomes derived from colorectal cancer cells exhibit the ability to augment the proliferation, migration, and angiogenesis of vascular endothelial cells

**Fig. 2:**
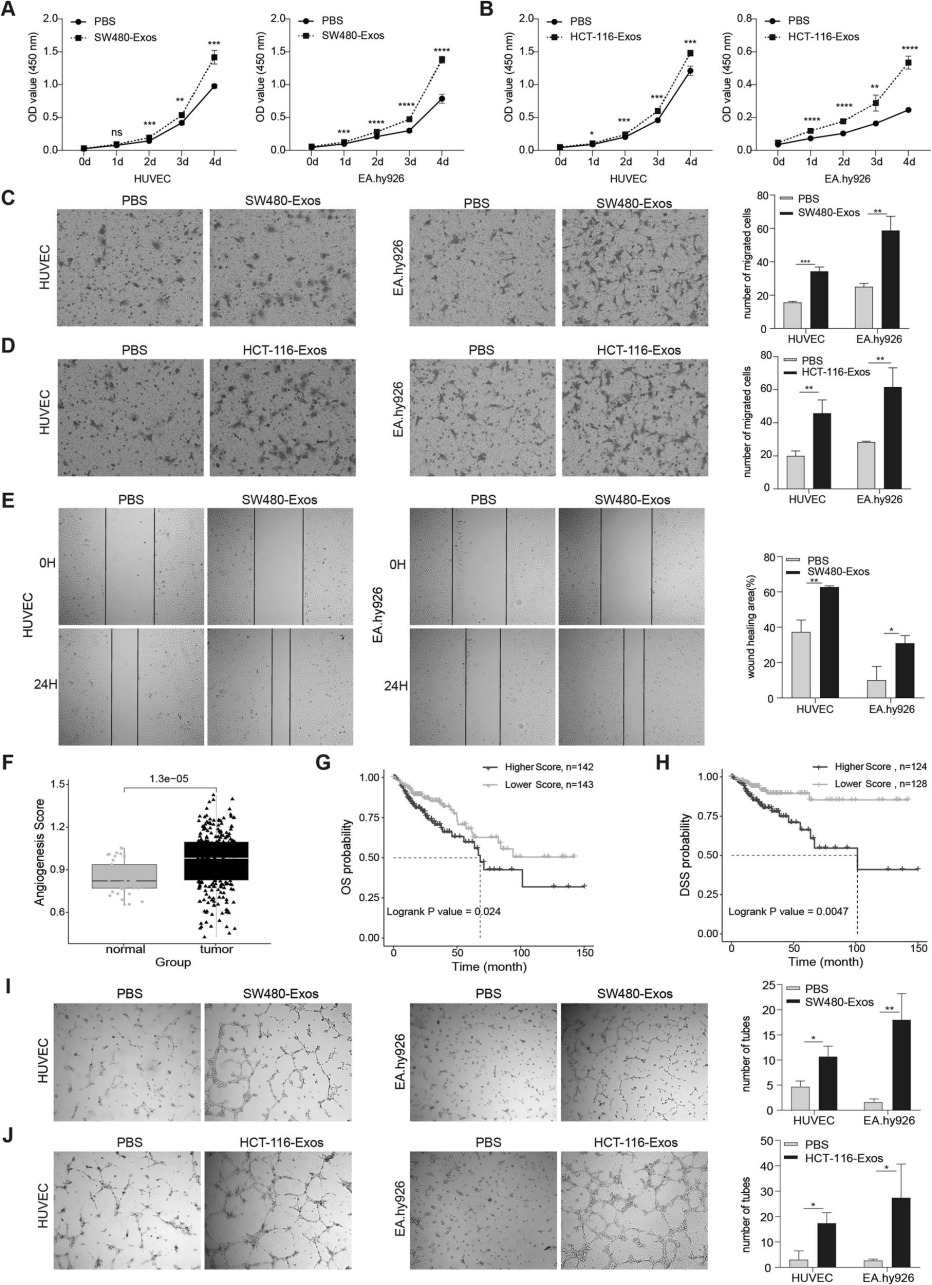
Exosomes derived from colorectal cancer cells exhibit the ability to augment the proliferation, migration, and angiogenesis of vascular endothelial cells. **A**, **B** CCK8 assay demonstrating the impact of exosomes derived from SW480 cells (**A**) and HCT-116 cells (**B**) on the proliferation of HUVEC and EA.hy926 cells. **C**, **D** Transwell assay depicting the influence of exosomes derived from SW480 cells (**C**) and HCT-116 cells (**D**) on the migration ability of HUVEC and EA.hy926 cells. **E** Cell wound-healing assay evaluating the effects of exosomes from SW480 on the migratory capacity of HUVEC (left) and EA.hy926 (right) cells. **F** Comparison of vascularity scores between paracarcinomatous and colorectal tissues. **G**, **H** Impact of high and low vasculature formation capacity scores on Overall Survival (OS) (**G**) and Progression-Free Survival (PFS) (**H**). **I**, **J** In vitro angiogenesis assay assessing the effect of exosomes from SW480 cells (**I**) and HCT-116 cells (**J**) on the angiogenic potential of HUVEC and EA.hy926 cells. (Two-tailed unpaired *t*-test or Mann–Whitney test. The Kaplan–Meier method was utilized to analyze survival curves, with significance evaluated using the log-rank test. *P < 0.05, **P < 0.01, ***P < 0.001, ****P < 0.0001).

To investigate the impact of tumor-derived exosomes on cancer progression, we isolated exosomes from SW480 and HCT-116 cells in complete medium and co-cultured them with vascular endothelial cells. Subsequently, a series of in vitro assays, including cell proliferation, migration, wound healing, and angiogenesis, were conducted to assess the effects of CRC-derived exosomes on vascular endothelial cells. The results revealed a significant increase in the proliferation of HUVEC and EA.hy926 cells following co-culture with exosomes released from SW480 and HCT-116 cells, compared to the PBS control group (Fig. 2A, B). Transwell migration assays (Fig. 2C, D) and wound-healing assays (Fig. 2E) demonstrated a notable enhancement in the migration of vascular endothelial cells after co-culture with exosomes. Moreover, our study compared the angiogenic capacity of CRC tissues with paracancerous tissues, revealing significantly higher angiogenesis scores in CRC tissues (Fig. 2F). Remarkably, CRC patients with high angiogenic capacity scores exhibited lower overall survival and progression-free survival rates compared to those with low scores (Fig. 2G, H). Additionally, in vitro angiogenesis assays (Fig. 2I, J) demonstrated that CRC-derived exosomes promoted the angiogenic ability of vascular endothelial cells. Hence, we conclude that exosomes derived from colorectal cancer cells can potentially facilitate tumor metastasis by influencing the proliferation and migration of vascular endothelial cells and promoting angiogenesis.

### The expression of miR-320d is elevated in colorectal cancer tissues and colorectal cancer serum exosomes, and miR-320d can be transported to endothelial cells via exosomes

**Fig. 3:**
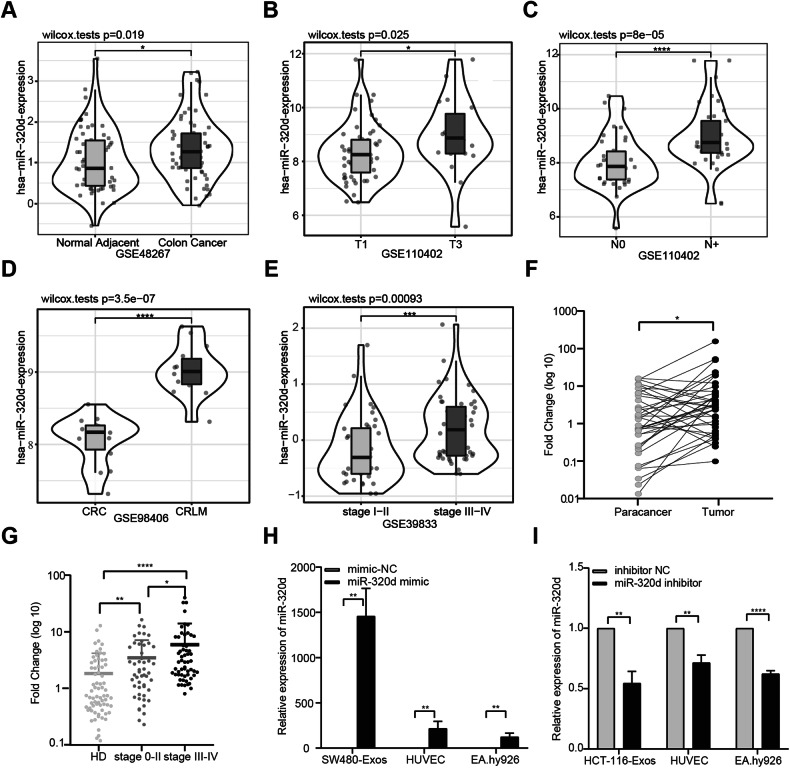
The expression of miR-320d is elevated in colorectal cancer tissues and colorectal cancer serum exosomes, and miR-320d can be transported to endothelial cells via exosomes. **A**–**D** Expression profile of miR-320d in CRC tissues and paracancerous tissues obtained from the GEO tissue database, and its correlation with TNM staging. **E** Expression level of miR-320d in CRC and HD serum exosomes from the GEO serum exosome database. **F** Comparison of miR-320d expression in CRC tissues and paracancerous tissues (*n* = 38). **G** Comparison of miR-320d expression in serum exosomes of HD and CRC patients (HD *n* = 70; CRC *n* = 108). **H** Expression of miR-320d in exosomes secreted by cells after overexpression of miR-320d in SW480 cells and subsequent incubation of exosomes with endothelial cells. **I** Expression of miR-320d in exosomes secreted by cells after knockdown of miR-320d in HCT-116 cells and subsequent incubation of exosomes with endothelial cells. (Two-tailed paired *t*-test, two-tailed unpaired *t*-test or Mann–Whitney test *P < 0.05, **P < 0.01, ****P < 0.0001).

Given our previous findings indicating significantly elevated miR-320d expression in serum exosomes from metastatic colorectal cancer patients, we hypothesized that exosomal miR-320d derived from colorectal cancer cells might contribute to endothelial cell proliferation, migration, and angiogenesis promotion. Through comprehensive analysis of GSE datasets, we initially observed a significant upregulation of miR-320d expression in CRC tissues compared to paracarcinoma tissues (Fig. 3A). Furthermore, miR-320d expression levels correlated significantly with CRC TNM stage, with higher levels detected in T3-stage patients compared to T1-stage patients, suggesting an association between miR-320d and tumor progression (Fig. 3B). Additionally, miR-320d expression was markedly elevated in CRC patients with lymph node metastasis and liver metastasis compared to those without metastasis, further suggesting a potential role in malignant metastasis (Fig. 3C, D). Notably, analysis of the GSE39833 dataset revealed significantly elevated levels of serum exosomal miR-320d in advanced CRC patients compared to early-stage patients (Fig. 3E). Subsequent confirmation of miR-320d expression in tissue and serum samples from resected colorectal cancer patients showed upregulation in cancer tissues compared to paracancerous tissues (Fig. 3F) and significantly higher levels in serum exosomes of colorectal cancer patients compared to healthy volunteers, with increasing tumor malignancy correlating with elevated miR-320d expression levels (Fig. 3G). Based on these findings, we focused our subsequent investigation on miR-320d. To explore the impact of exosomal miR-320d on endothelial cell proliferation, migration, and angiogenesis, we first assessed baseline miR-320d expression levels in colorectal cancer cells, including SW480, HCT-116, LS174T, and LOVO (Supplementary Fig. 1B). Subsequently, we transfected miR-320d mimic/mimic-NC in SW480 cells and miR-320d inhibitor/inhibitor NC in HCT-116 cells. Following this, we isolated exosomes from the cell medium and co-cultured them with vascular endothelial cells. Evaluation of miR-320d expression levels in released exosomes revealed significant upregulation or downregulation induced by miR-320d mimics/inhibitors. Furthermore, after incubating endothelial cells with these exosomes for 12 h, we observed similar changes in miR-320d expression levels in HUVEC and EA.hy926 cells as those in exosomes (Fig. 3H, I). These results suggest that miR-320d can be transported to endothelial cells via exosomes, potentially influencing the interaction between colorectal cancer cells and vascular endothelial cells.

### In vitro experiments to validate the effects of exosomal miR-320d on the proliferation, migration and angiogenic capacity of vascular endothelial cells and the JAK2/STAT3 pathway

**Fig. 4:**
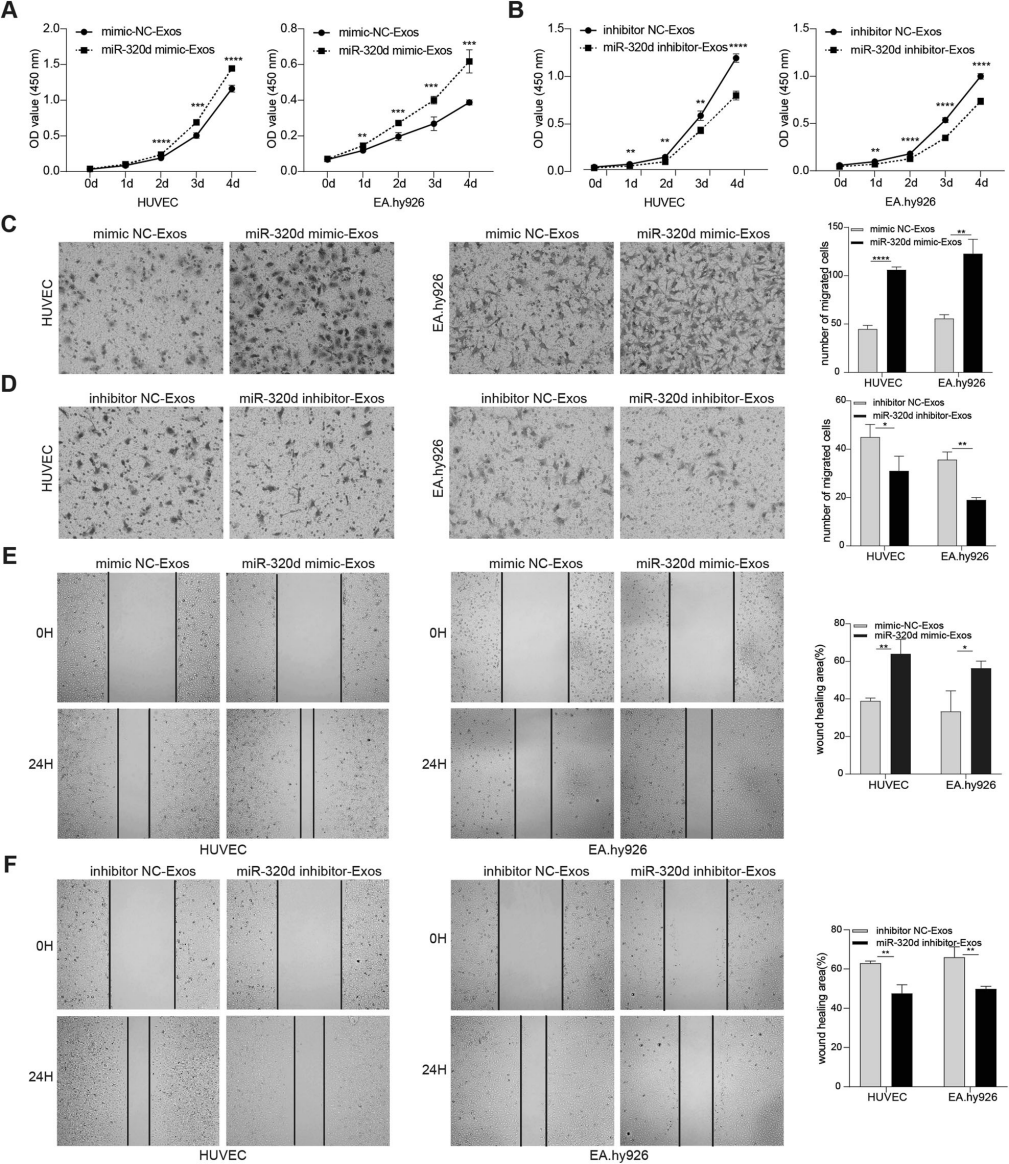
Exosomal miR-320d enhances vascular endothelial cell proliferation and migration. **A**, **B** CCK8 assay to assess the impact of miR-320d mimics-Exos (**A**) and exosomes containing miR-320d inhibitor (**B**) on the proliferative capacity of vascular endothelial cells. **C**, **D** Transwell assay demonstrating the effects of miR-320d mimics-Exos (**C**) and exosomes containing miR-320d inhibitor (**D**) on the migration ability of vascular endothelial cells. **E**, **F** Cell wound-healing assay evaluating the effects of miR-320d mimics-Exos (**E**) and exosomes containing miR-320d inhibitor (**F**) on the migration ability of HUVEC (left) and EA.hy926 (right) cells. (Two-tailed unpaired *t*-test or Mann–Whitney test *P < 0.05, **P < 0.01, ***P < 0.001, ****P < 0.0001).

**Fig. 5:**
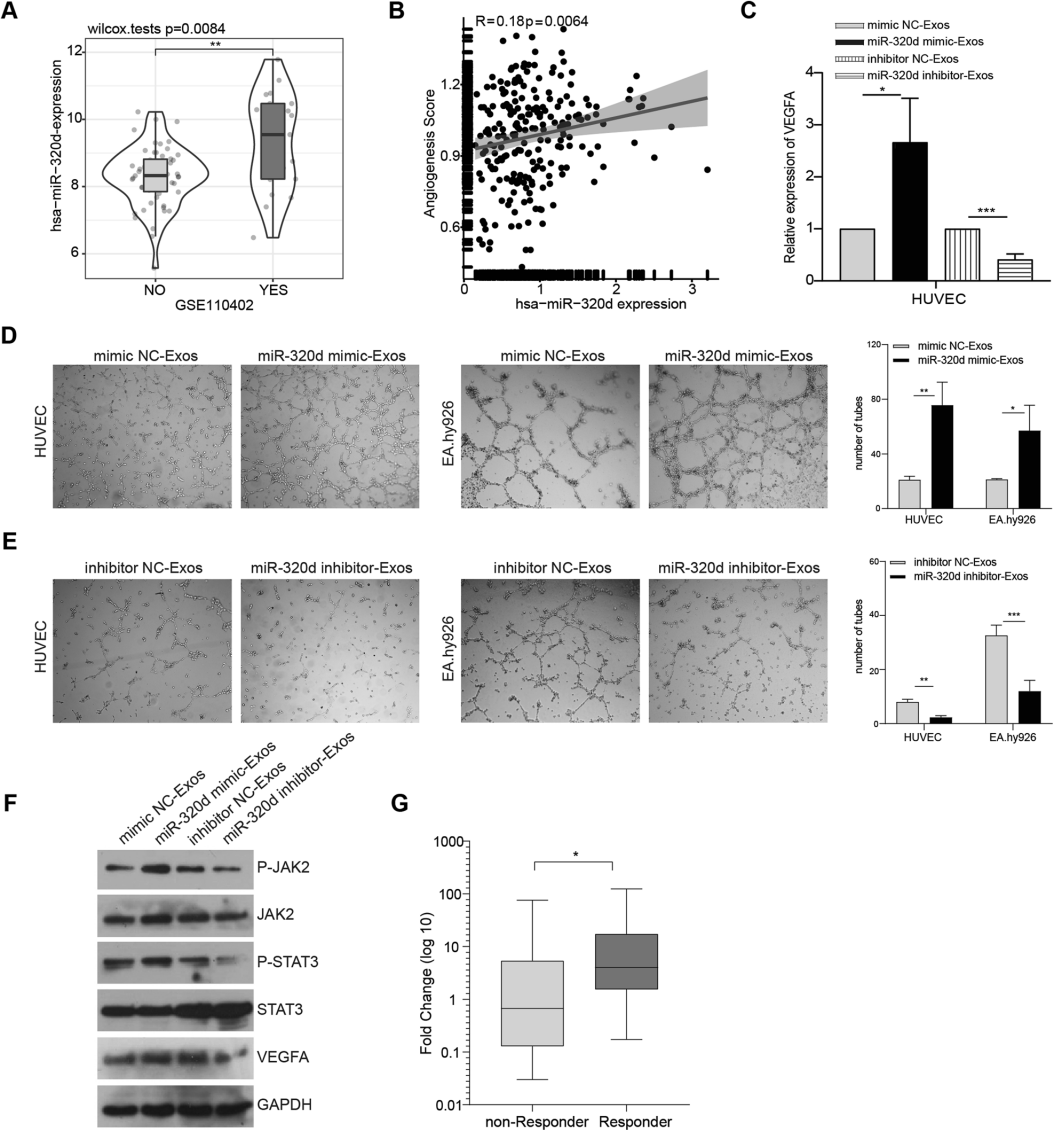
In vitro experiments to validate the effect of exosomal miR-320d on vascular endothelial angiogenic capacity and on the JAK2/STAT3 pathway. **A** Analysis of GSE dataset revealing high expression of miR-320d in patients with lymphovascular invasion. **B** Correlation analysis of miR-320d expression with scores of angiogenic capacity. **C** qPCR assay showing the effect of miR-320d mimics /inhibitor-Exos on VEGFA expression in vascular endothelial cells. **D**, **E** Impact of miR-320d mimics /inhibitor-Exos on the angiogenic capacity of vascular endothelial cells. **F** Effect of miR-320d mimics/inhibitor-Exos on JAK2/STAT3/VEGFA expression in HUVEC. **G** Role of serum exosomal miR-320d in predicting the efficacy of bevacizumab chemotherapy in CRC non-responder *n* = 23; Responder *n* = 14. (Two-tailed unpaired *t*-test or Mann–Whitney test *P < 0.05, **P < 0.01, ***P < 0.001).

We extensively investigated the impact of exosomal miR-320d derived from colorectal cancer cells on vascular endothelial cells through in vitro experiments. Our experimental findings revealed that miR-320d mimics-Exos obtained from SW480 cells exerted a noticeable promotional effect on vascular endothelial cells, significantly enhancing their proliferation and migration abilities. Conversely, miR-320d inhibitor-Exos derived from HCT-116 cells exhibited a substantial inhibitory effect, markedly attenuating the proliferation and migration abilities of vascular endothelial cells (Fig. 4A–F). Additionally, we explored the GSE110402 dataset and observed that CRC patients with high miR-320d expression were more prone to vascular invasion (Fig. 5A), and the expression level of miR-320d positively correlated with the angiogenic capacity in CRC patients (Fig. 5B). Subsequently, we evaluated the expression levels of VEGFA RNA and protein in HUVEC cells after incubation with exosomes, revealing that exosomal miR-320d promoted VEGFA expression levels in vascular endothelial cells (Fig. 5C). Furthermore, in vitro angiogenesis experiments confirmed that miR-320d mimics-Exos significantly enhanced the angiogenic capacity of vascular endothelial cells in SW480 cells, while miR-320d inhibitor-Exos notably inhibited the angiogenic capacity of HCT-116 cells (Fig. 5D, E). These results elucidate the critical role of exosomal miR-320d in CRC and its interaction with vascular endothelial cells. Considering relevant literature, we explored the phosphorylation of JAK2/STAT3 proteins in HUVEC cells after incubation with miR-320d mimics-Exos and miR-320d inhibitor-Exos to delve deeper into the underlying mechanism. Our experimental findings demonstrated that miR-320d mimics-Exos promoted the phosphorylation of JAK2 and STAT3 proteins in HUVEC cells, while miR-320d inhibitor-Exos inhibited this phosphorylation process (Fig. 5F). Notably, bevacizumab, a drug commonly used in clinical practice for CRC treatment, specifically binds to vascular endothelial growth factor, blocking its interaction with vascular endothelial growth factor receptor and subsequently inhibiting the signaling pathway of angiogenesis. This inhibition impedes tumor neovascularization, suppresses tumor cell growth, and exerts an anti-tumor effect. To further explore the efficacy of bevacizumab in CRC patients, we assessed the expression levels of serum exosomal miR-320d in patients before treatment with this drug and analyzed its impact on therapeutic outcomes. Our analysis revealed that CRC patients with high miR-320d expression exhibited significantly better response to bevacizumab compared to those with low miR-320d expression (Fig. 5G).

### Exosomal miR-320d promotes tumor growth, metastasis, and angiogenesis in vivo

**Fig. 6:**
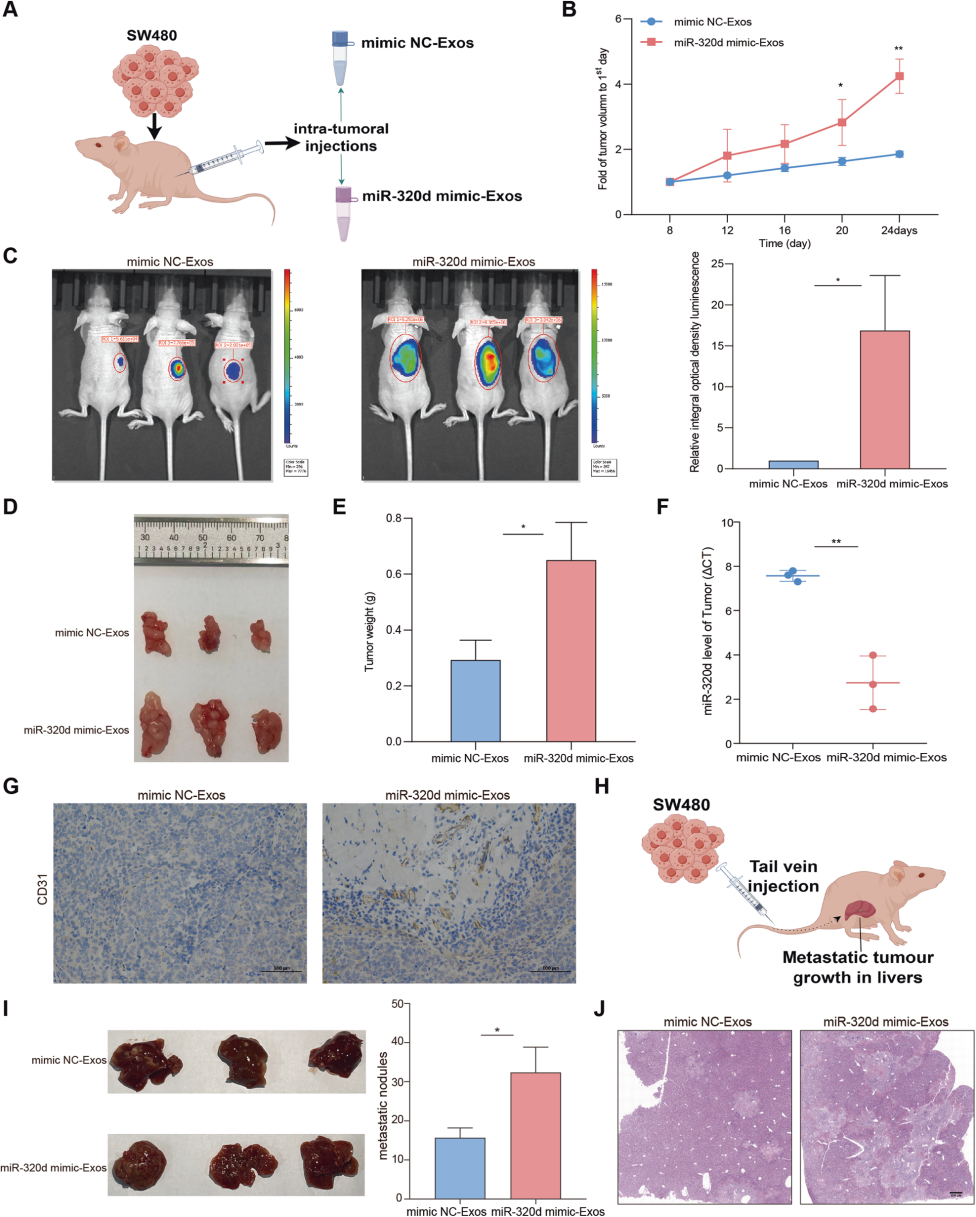
Exosomal miR-320d promotes tumor growth, metastasis, and angiogenesis in vivo. **A** Nude mouse xenograft tumor model. **B**–**E** Analysis of growth rate (**B**), luciferase signal intensity (**C**), volume (**D**), and weight (**E**) of nude mouse graft tumors (*n* = 6). **F** Expression levels of miR-320d in nude mouse transplant tumors. **G** Expression level of CD31 in transplanted tumors of nude mice via IHC. **H** Nude mice liver metastasis model. **I**, **J** HE staining to observe liver metastasis nodules in nude mice (*n* = 6). (Two-tailed unpaired *t*-test or Mann–Whitney test *P < 0.05, **P < 0.01, ***P < 0.001, ****P < 0.0001).

To delve into the mechanism of exosomal miR-320d in vivo, we established a tumor xenograft model in nude mice using SW480 cells (Fig. 6A). Throughout tumor growth, the experimental group of nude mice received injections of miR-320d mimics-Exos or mimics NC-Exos every 4 days. The results unveiled a significant acceleration in the growth rate and weight of tumors in mice treated with miR-320d mimics-Exos compared to the control group (Fig. 6B–E). Additionally, to confirm the successful establishment of the tumor xenograft model, we assessed the expression level of miR-320d within the tumor (Fig. 6F). Immunohistochemistry results demonstrated that exosomal miR-320d enhanced the expression of CD31 and VEGF within the tumor (Fig. 6G and Supplementary Fig. 1C). Additionally, to observe the changes of angiogenic indexes in vivo, Matrigel plug assay was carried out, and the dissected Matrigel plugs were stained with HE. The results showed that tumors in mice treated with miR-320d mimics-Exos showed significantly more angiogenesis compared to the control group (Supplementary Fig. 1D). Subsequently, we established a tumor metastasis model via tail vein injection, utilizing SW480 cells pretreated with miR-320d mimics-Exos and mimics NC-Exos (Fig. 6H). The findings indicated a significant increase in liver nodules in mice belonging to the miR-320d mimics-Exos group compared to those in the mimics NC-Exos group (Fig. 6I, J). These results suggest that exosomal miR-320d potentially contributes to the promotion of colorectal cancer angiogenesis and metastasis in vivo.

### GNAI1 is a functional target of miR-320d

**Fig. 7:**
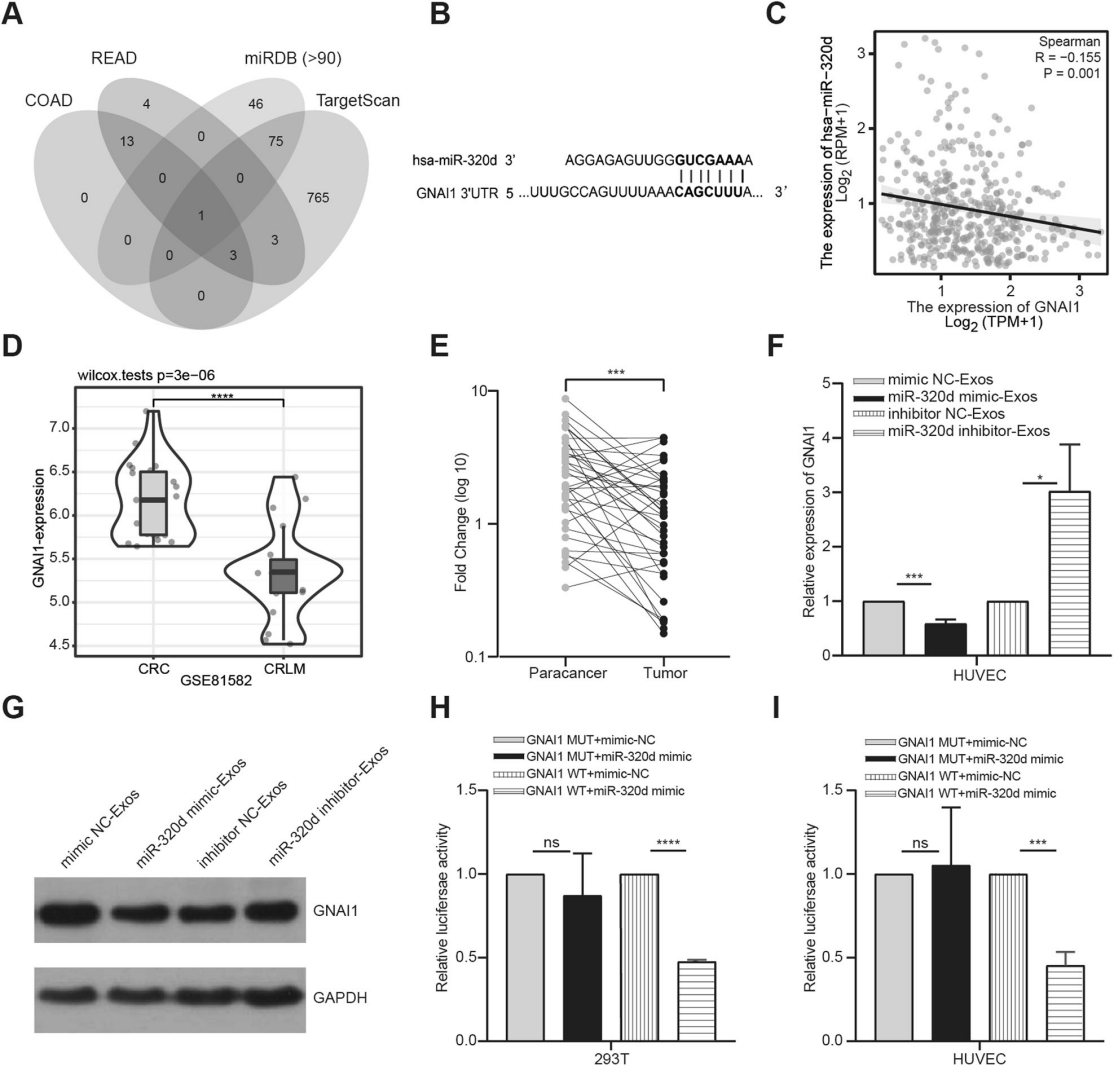
GNAI1 is a functional target of miR-320d. **A** Predicted target genes in TCGA, miRDB, and Targetscan databases. **B** GNAI1 and miR-320d binding sites. **C** TCGA database showing the negative correlation between GNAI1 expression and miR-320d expression. **D** GSE dataset illustrating the low expression of GNAI1 in liver metastatic CRC patients. **E** Expression levels of GNAI1 in CRC tissues and paracancerous tissues (*n* = 38). **F** qRT-PCR for GNAI1 expression in endothelial cells after overexpression of miR-320d and knockdown of miR-320d. **G** Western Blots detection of GNAI1 expression in HUVEC after overexpression of miR-320d and knockdown of miR-320d. **H**, **I** Dual-luciferase reporter assay for the regulation of GNAI1 by miR-320d; 293T cells (**H**); HUVEC (**I**). (Two-tailed paired *t*-test, two-tailed unpaired *t*-test or Mann–Whitney test. Spearman correlation analysis was employed to evaluate the degree of linear relationship between miR-320d and GNAI1. *P < 0.05, **P < 0.01, ****P < 0.0001).

As described in the materials and methods, to elucidate the potential downstream targets of miR-320d, we performed Spearman correlation analysis on the TCGA colorectal cohort to identify mRNAs negatively correlated with miR-320d. Subsequently, we predicted downstream genes of miR-320d using the miRDB and TargetScan databases. Finally, we intersected the results from the above analyses to obtain the final target gene GNAI1 (Fig. 7A). Subsequent analysis based on the TargetScan database confirmed the presence of a miR-320d base-paired region in the 3’UTR of GNAI1 (Fig. 7B). Furthermore, analysis using the TCGA database demonstrated a negative correlation between the expression levels of miR-320d and GNAI1 (Fig. 7C), in line with the regulatory mechanism of miRNAs. Therefore, we selected GNAI1 for further experimental validation. Building upon the database analysis results, we observed that CRC patients who developed liver metastases exhibited significantly lower expression of GNAI1 compared to those who did not develop liver metastases (Fig. 7D). Additionally, utilizing clinical tissue samples, qRT-PCR results revealed significantly lower expression of GNAI1 in CRC tissues compared to paracancerous tissues (Fig. 7E). To investigate whether GNAI1 is a target gene of miR-320d, we conducted intracellular validation. Initially, we observed that both RNA and protein levels of GNAI1 were significantly reduced in HUVEC cells cultured with miR-320d mimics-Exos. Conversely, the expression level of GNAI1 significantly increased when cultured with exosomal miR-320d inhibitor (Fig. 7F, G). Subsequently, we cloned the GNAI1 3’UTR wild and mutant sequences that bind complementarily to miR-320d into luciferase reporter vectors. After co-transfecting these vectors with miR-320d into 293T and HUVEC cells, we found that the luciferase activity of the GNAI1 3’UTR mutant group was significantly higher than that of the GNAI1 3’UTR wild-type group (Fig. 7H, I). This result confirmed GNAI1 as a downstream target gene of miR-320d.

### Silencing GNAI1 enhances vascular endothelial cell proliferation, migration and angiogenesis

**Fig. 8:**
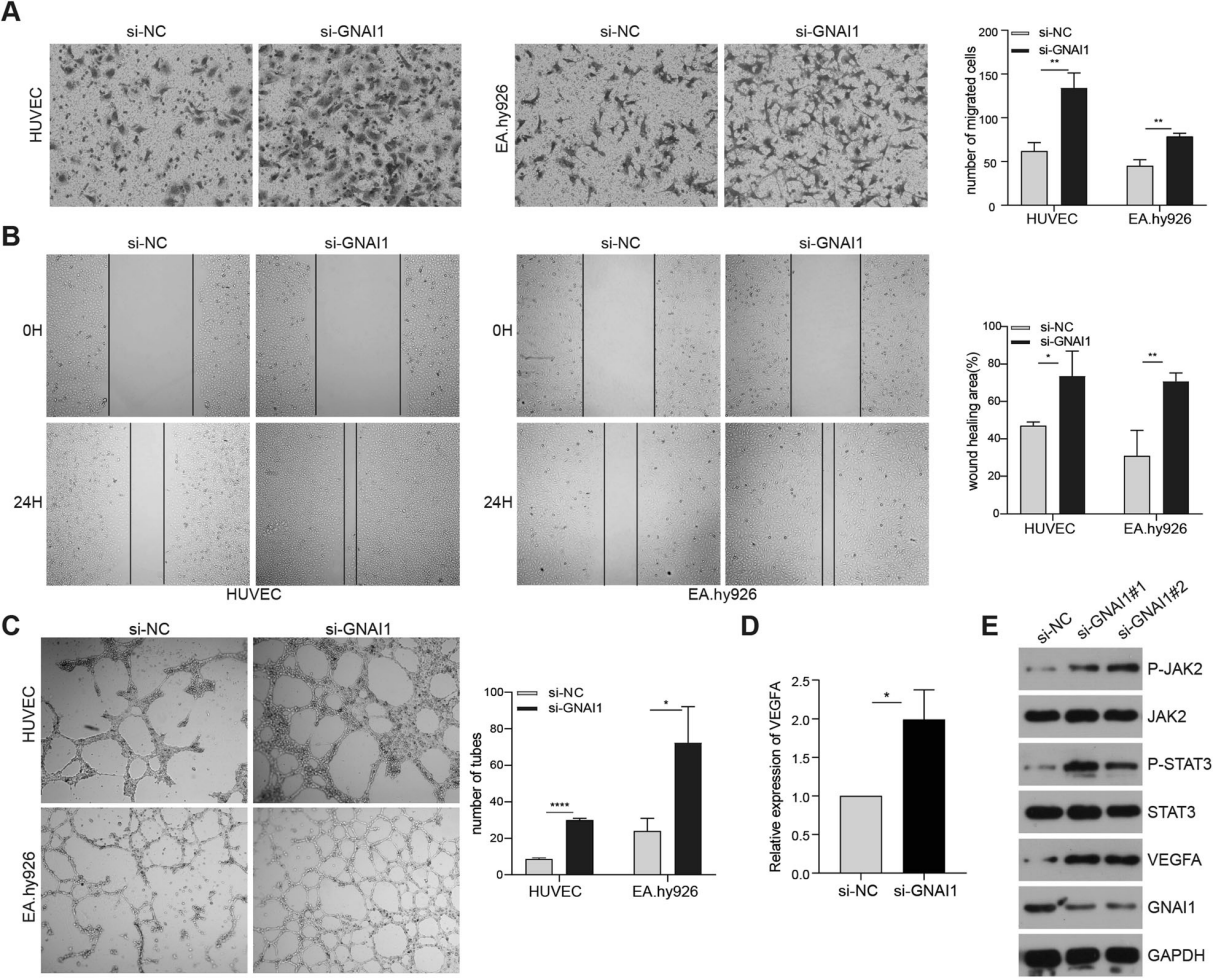
Silencing GNAI1 enhances vascular endothelial cell proliferation, migration and angiogenesis. **A**, **B** Transwell (**A**) and cell wound-healing assay (**B**) validating the effect of si-GNAI1 on the metastatic capacity of HUVEC (left) and EA.hy926 (right) cells. **C** In vitro angiogenesis assay confirming the effect of si-GNAI1 on vascular endothelial cell angiogenic capacity. **D** qPCR detection of VEGFA expression level in vascular endothelial cells after si-GNAI1. **E** Western Blots detection of phosphorylation level of JAK2/STST3 with VEGFA expression level in HUVEC after si-GNAI1. (Two-tailed unpaired *t*-test or Mann–Whitney test *P < 0.05, **P < 0.01, ***P < 0.001, ****P < 0.0001).

To explore the involvement of GNAI1 in the regulation of endothelial cell migration and angiogenic capacity, we synthesized small interfering RNA and transfected vascular endothelial cells to silence intracellular GNAI1 expression. Subsequently, we evaluated the expression of GNAI1 in vascular endothelial cells using qRT-PCR after GNAI1 interference (Supplementary Fig. 1E). Following this, through transwell and wound-healing experiments, we observed a significant enhancement in the migration ability of vascular endothelial cells after GNAI1 interference compared to the control group (Fig. 8A, B). Furthermore, our in vitro angiogenesis experiment validated a notable increase in the angiogenic capacity of vascular endothelial cells after GNAI1 silencing (Fig. 8C). Moreover, we delved deeper into the impact of silencing GNAI1 on the JAK2/STAT3 signaling pathway in vascular endothelial cells. Our findings unveiled that silencing GNAI1 promoted the phosphorylation of JAK2/STAT3 proteins, subsequently resulting in the upregulation of VEGFA expression (Fig. 8D, E). This effect mirrored that observed after incubation with exosomal miR-320d.

### Exosomal miR-320d promotes angiogenesis in colorectal cancer by silencing GNAI1 affecting the JAK2/STAT3 signaling pathway

**Fig. 9:**
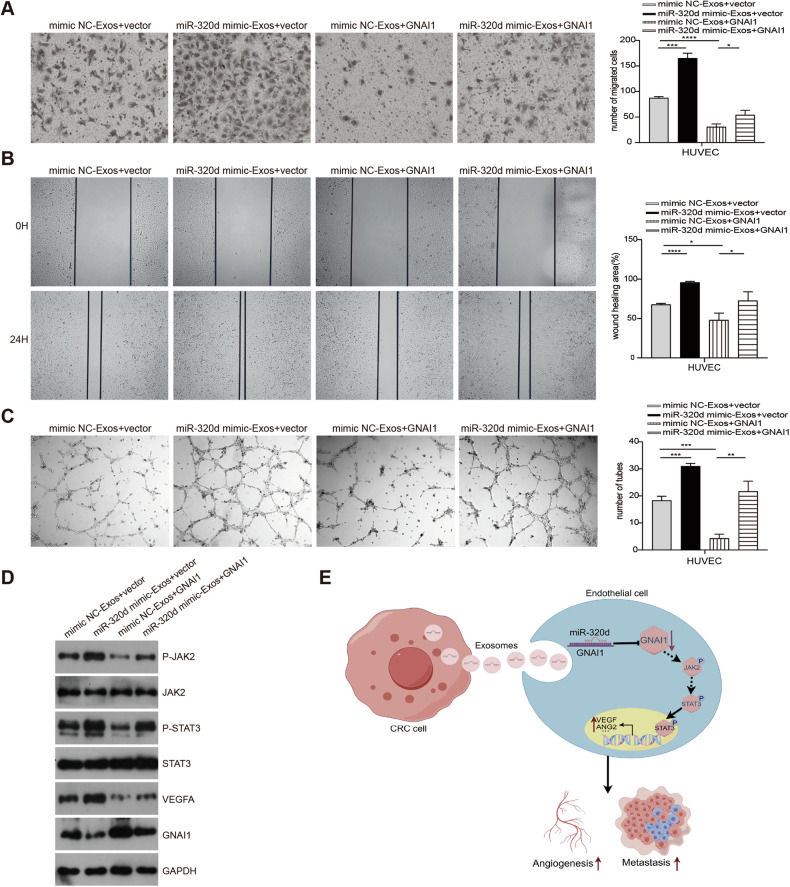
Exosomal miR-320d promotes angiogenesis in colorectal cancer by silencing GNAI1 affecting the JAK2/STAT3 signaling pathway. **A**, **B** Transwell (**A**) and cell wound-healing assay (**B**) verifying the effect of exosomal miR-320d targeting GNAI1 on HUVEC migration capacity. **C** In vitro angiogenesis assay confirming the effect of exosomal miR-320d targeting GNAI1 on HUVEC angiogenic capacity. **D** Western Blots detection of the effect of exosomal miR-320d-targeted GNAI1 on the phosphorylation of JAK2/STAT3 and VEGFA expression levels in HUVEC. **E** Schematic of the working model. (Two-tailed unpaired *t*-test or Mann–Whitney test *P < 0.05, **P < 0.01, ***P < 0.001, ****P < 0.0001).

We investigated the impact of exosomal miR-320d on the migration and angiogenic capacity of vascular endothelial cells by silencing GNAI1 through in vitro experiments. Initially, we treated vascular endothelial cells with miR-320d mimics-Exosomes, mimics NC-Exosomes, and GNAI1 overexpression. We validated the changes in GNAI1 expression levels across different groups using western blotting (Fig. 9D) and PCR detection (Supplementary Fig. 1F) before conducting transwell, wound-healing, and angiogenesis experiments. Our results revealed that miR-320d mimics-Exos promoted the migration and angiogenic capacity of HUVECs, whereas overexpression of GNAI1 inhibited these processes compared to the control group. Notably, overexpression of GNAI1 reversed the migration and angiogenic capacity induced by miR-320d mimics-Exos in HUVECs (Fig. 9A–C). Furthermore, changes induced by miR-320d mimics-Exos in p-JAK2, p-STAT3, and VEGFA expression were similarly reversed by GNAI1 overexpression, as confirmed by western blot experiments (Fig. 9D). These findings suggest that exosomal miR-320d promotes the phosphorylation and nuclear translocation of JAK2/STAT3 proteins, enhances VEGFA expression by targeting GNAI1, thereby augmenting the angiogenic capacity of vascular endothelial cells (Fig. 9E).

## Discussion

In this study, we have demonstrated that exosomal miR-320d derived from colorectal cancer cells enhances angiogenesis in vascular endothelial cells and promotes cancer cell metastasis by downregulating GNAI1 expression, enhancing JAK2/STAT3 protein phosphorylation, and increasing VEGFA expression.

Numerous studies have reported that tumor-derived exosomal miRNAs can regulate angiogenesis and facilitate tumor metastasis by targeting specific genes or signaling pathways. For instance, in gastric cancer, miR-519a-3p contained in exosomes was internalized by intrahepatic macrophages, leading to macrophage M2-like polarization, thereby accelerating gastric cancer liver metastasis through angiogenesis induction and the promotion of pre-metastatic niche formation in the liver [12]. Furthermore, exosomal miR-205 derived from ovarian cancer has been found to promote angiogenesis both in vitro and in vivo by regulating the PTEN-AKT pathway, consequently facilitating ovarian cancer metastasis [13]. Additionally, studies have indicated that miR-320d plays a role in regulating gastric cancer progression by modulating downstream FoxM [14], while exosomal lncRNA LINC00662 has been shown to promote non-small cell lung cancer progression by regulating the miR-320d/E2F1 axis [15]. However, miR-320d does not absolutely promote the progression of cancer. It has been reported in the literature that overexpression of miR-320d can inhibit the migration, invasion, proliferation and epithelial mesenchymal transition (EMT) of EGFR-positive CRC cells. miR-320d affects the PI3K/AKT signaling pathway through the modulation of TUSC3 expression to inhibit the progression of EGFR-positive CRC cells [16]. In summary, the role of miR-320d in CRC is not single and absolute but exhibits a complex dual regulatory mechanism. This duality may arise from the interactions between different cell types, microenvironments and signaling pathways.

The G protein subunit alpha i1 (GNAI1) acts as a suppressor of adenylate cyclase, thereby regulating cAMP levels and exerting a significant influence on tumor progression across various cancer types. Previous studies have demonstrated that the expression level of GNAI1 correlates with aggressive characteristics and unfavorable prognosis in gastric cancer [17] and hepatocellular carcinoma [18]. Moreover, reduced expression of GNAI1 has been notably associated with human colitis-associated colorectal cancer (CAC) [19]. These findings underscore the critical role of GNAI1 in tumor growth, invasion, and metastasis.

In tumor biology, aberrantly activated STAT3 emerges as a pivotal oncogenic factor, promoting cell proliferation, survival, invasion, and metastasis. Activated STAT3 can stimulate angiogenesis in tumors through various mechanisms. Initially, it induces endothelial cell proliferation and migration, thereby facilitating angiogenesis. For example, the IL-6/STAT3 pathway has been identified as a promoter of angiogenesis in hepatocellular carcinoma by enhancing endothelial cell migration and angiogenesis [20]. Furthermore, STAT3 regulates the transcription and expression of vascular endothelial growth factor (VEGF), a crucial pro-angiogenic factor that promotes angiogenesis by facilitating endothelial cell proliferation and vascular lumen formation. Overexpression of KPNA2, for instance, enhances STAT3 phosphorylation, subsequently upregulating VEGFA and angiopoietin 2 (ANGPT2) to augment angiogenesis [21]. Additionally, loss of GNAI1 leads to high basal levels of GP130 and TAK1-TABl interaction, which are likely responsible for high basal activation of NF-kB and STAT3. Knockout GNAI1 and GNAI3 in BMDSCs cells can induce phosphorylation of JAK2 and STAT3, promoting colitis-associated tumors in mice [19]. Moreover, CALM2 has been found to stimulate proliferation, migration, invasion, and angiogenesis of HUVECs, as well as M2 polarization of THP1 cells, through the JAK2/STAT3/HIF-1/VEGFA signaling pathway, thereby facilitating gastric cancer metastasis [22]. This study further validates the role of GNAI1 in promoting angiogenesis in colorectal cancer through its influence on STAT3 activity.

Our database analysis revealed that patients with CRC exhibit higher scores indicating their ability to form blood vessels. Furthermore, emerging research suggests that benign tumors, characterized by slow cell division and growth, tend to demonstrate relatively low vascular density. In contrast, malignant tumors, which undergo rapid cell proliferation and growth, require increased oxygen and nutrient supply, leading to a significant rise in blood vessel density [23, 24]. Consequently, inhibiting angiogenesis around malignant tumors has become a common strategy for treating solid tumors. Presently, the primary clinically employed anti-angiogenic agent is bevacizumab. By binding to VEGFA, bevacizumab impedes the generation and growth of new blood vessels around tumors, thereby inhibiting the interaction between VEGFA and its receptor [25]. Our investigative study discovered that among colorectal cancer patients, those exhibiting high expression of serum exosome miR-320d demonstrated enhanced efficacy in response to bevacizumab treatment compared to those with low expression. This finding may open up new therapeutic avenues for the clinical treatment of metastatic colorectal cancer patients.

## Supplementary information

Supplementary legends

Original western blots

Supplementary FIG 1

## Data Availability

The original contributions presented in the study are included in the article/Supplementary Material, further inquiries can be directed to the corresponding author.
