# Supplementary material for: Exosomal miR-320d promotes angiogenesis and colorectal cancer metastasis via targeting GNAI1 to affect the JAK2/STAT3 signaling pathway
Source: Cell Death Dis. 2024 Dec 18;15(12):913. doi: 10.1038/s41419-024-07297-y (PMC11655962; doi:10.1038/s41419-024-07297-y)
Supplement: Supplementary file 1 — Supplementary legends [file 41419_2024_7297_MOESM1_ESM.docx]

**Supplementary Figure 1**

**(A)** Cell wound-healing assay verifying the effects of HCT-116 cell-derived exosomes on the migratory capacity of HUVEC (left) and EA.hy926 (right) cells. **(B)** The baseline expression levels of miR-320d in colorectal cancer cells. **(C)** Expression levels of vascular endothelial growth factor in xenograft tumors of nude mice by IHC. **(D)** Matrigel plug assay showed that exosomal miR-320d promotes angiogenesis in vivo (n=6). HE staining were carried out on the dissected Matrigel plugs. **(E)** The expression of GNAI1 in vascular endothelial cells using qRT-PCR after GNAI1 interference. **(F)** qPCR detection confirmed changes in GNAI1 expression levels across different groups. (Two-tailed unpaired t test or Mann-Whitney test *P < 0.05, **P < 0.01, ***P < 0.001, ****P < 0.0001.)
