# Supplementary figures and images for: Exosomal miR-320d promotes angiogenesis and colorectal cancer metastasis via targeting GNAI1 to affect the JAK2/STAT3 signaling pathway
Source: Cell Death Dis. 2024 Dec 18;15(12):913. doi: 10.1038/s41419-024-07297-y (PMC11655962; doi:10.1038/s41419-024-07297-y)

Figure 1

C

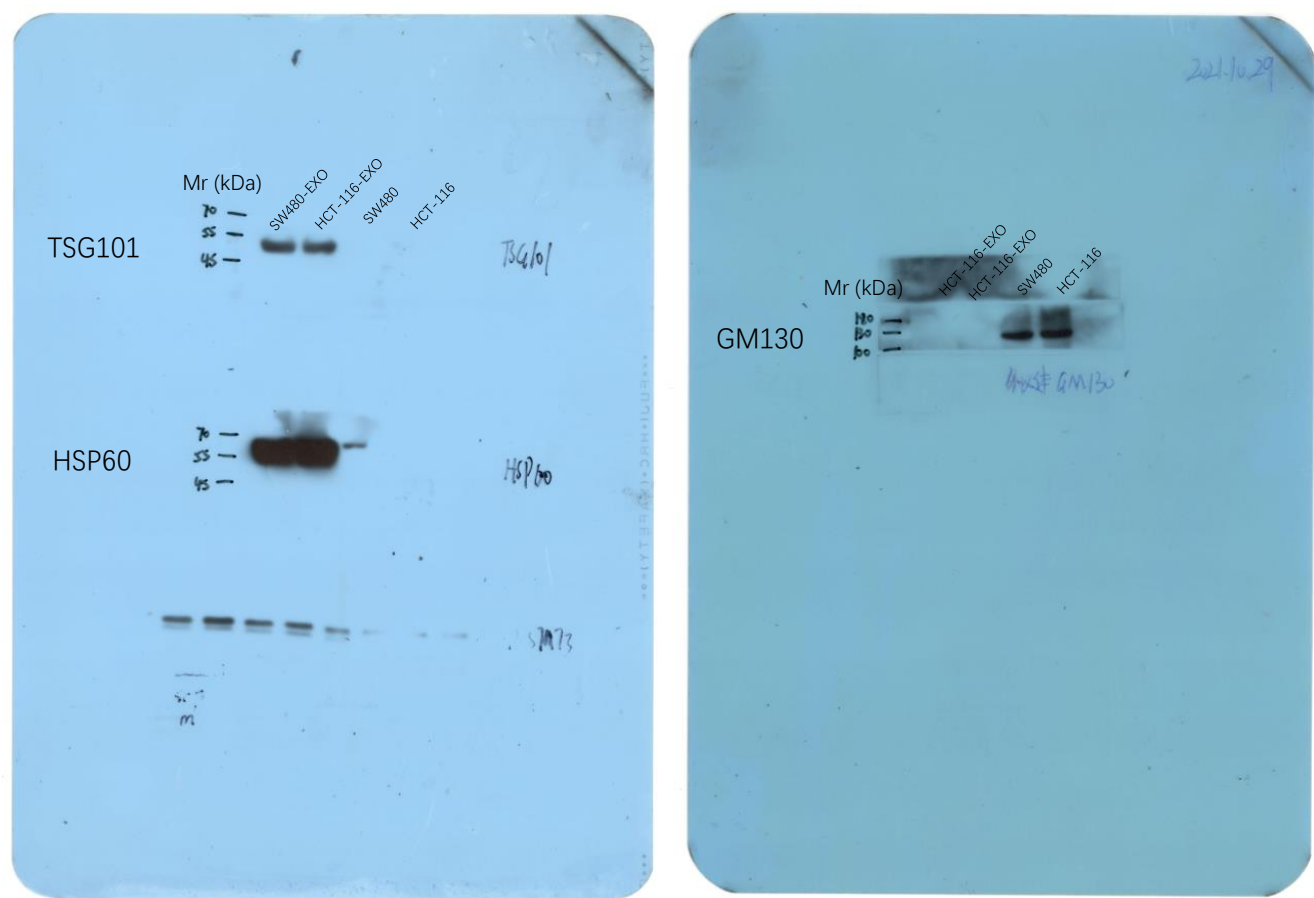

Figure 5

F

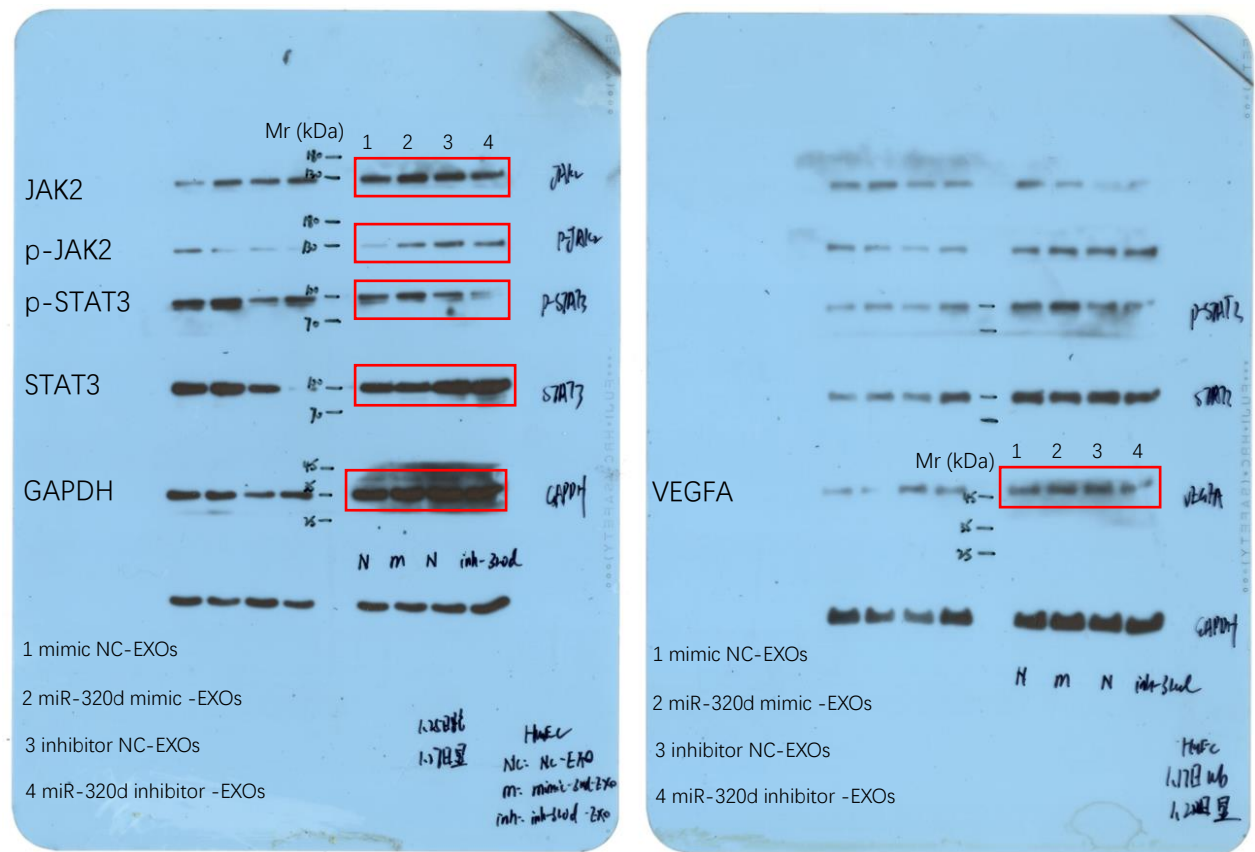

Figure 7

G

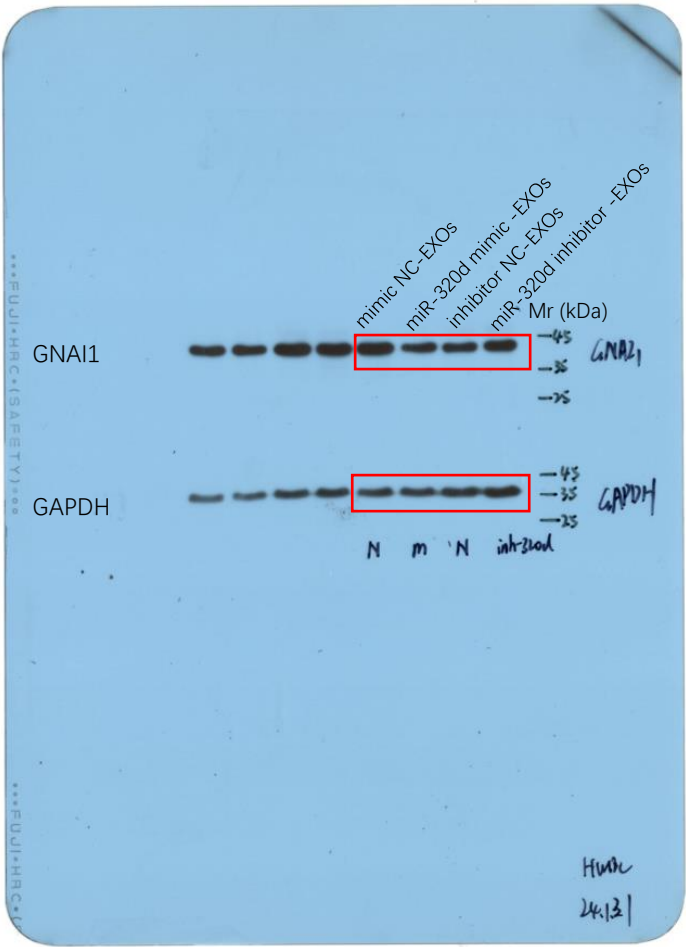

Figure 8

E

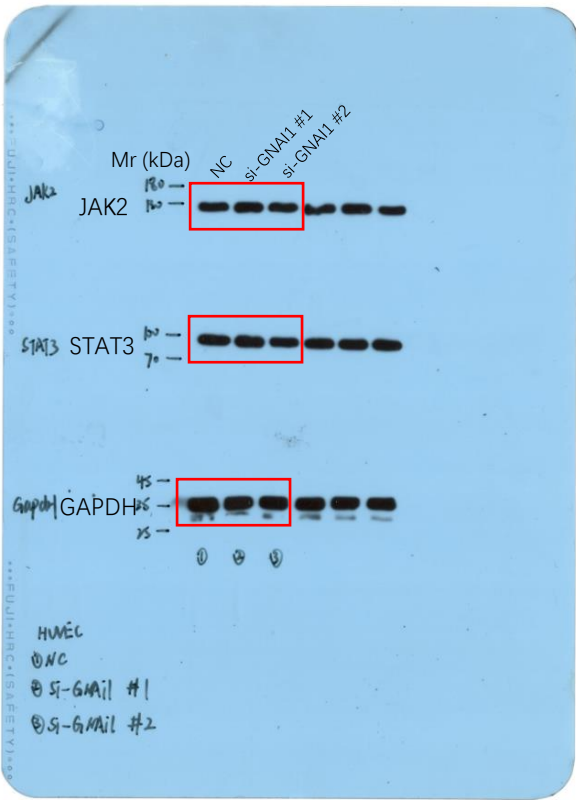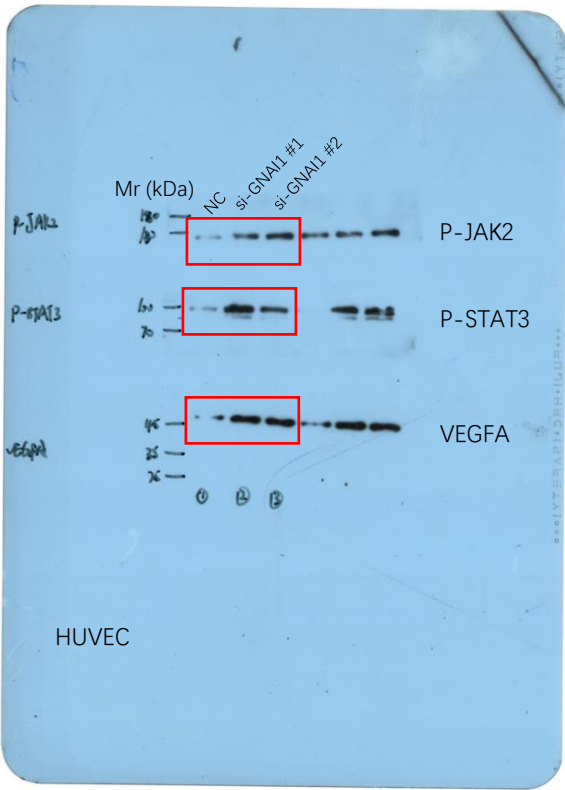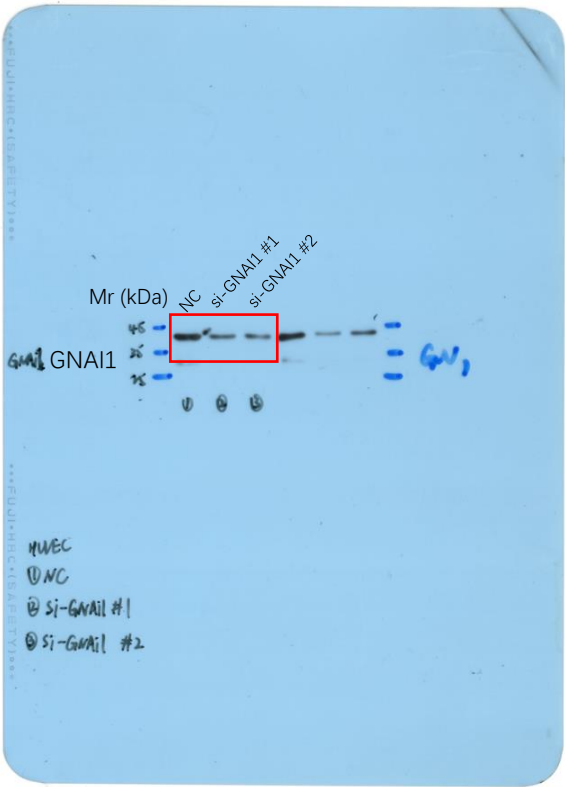

Figure 9

D

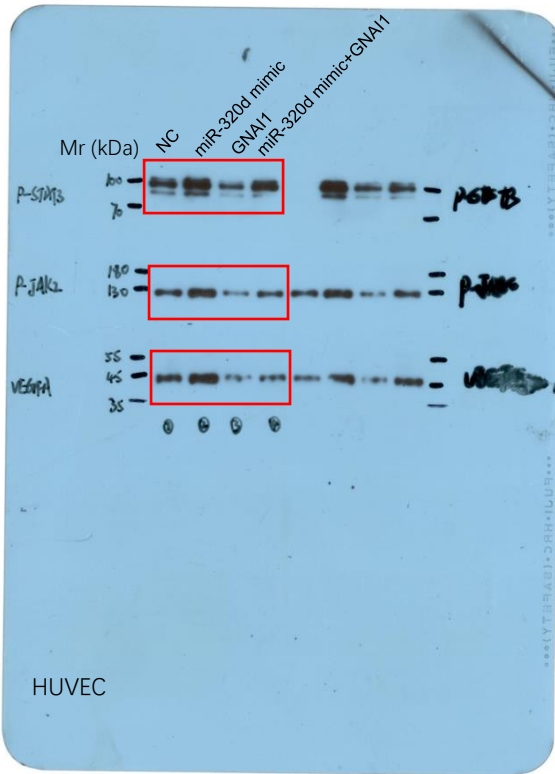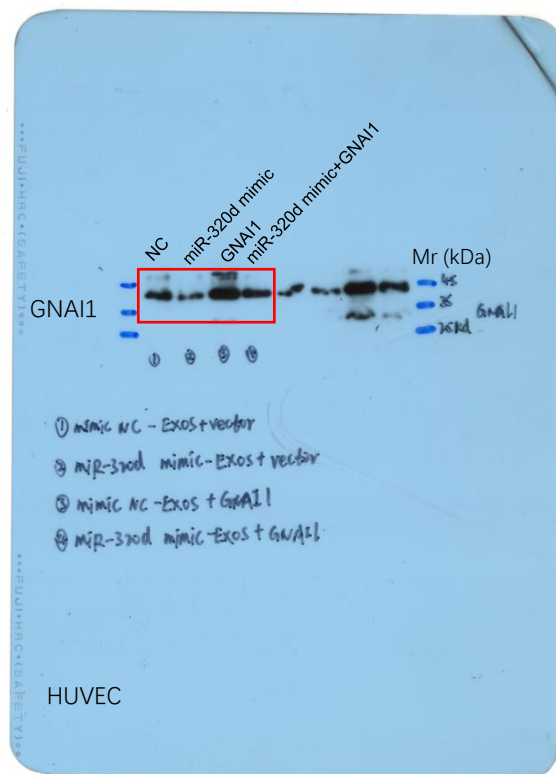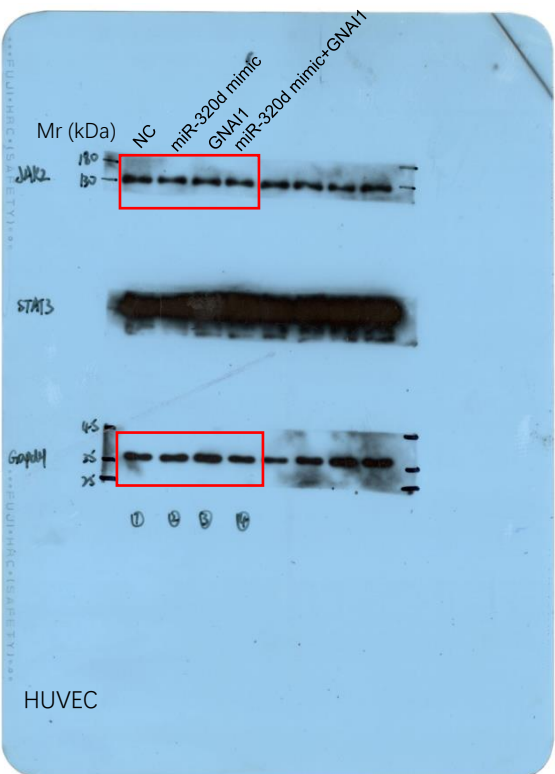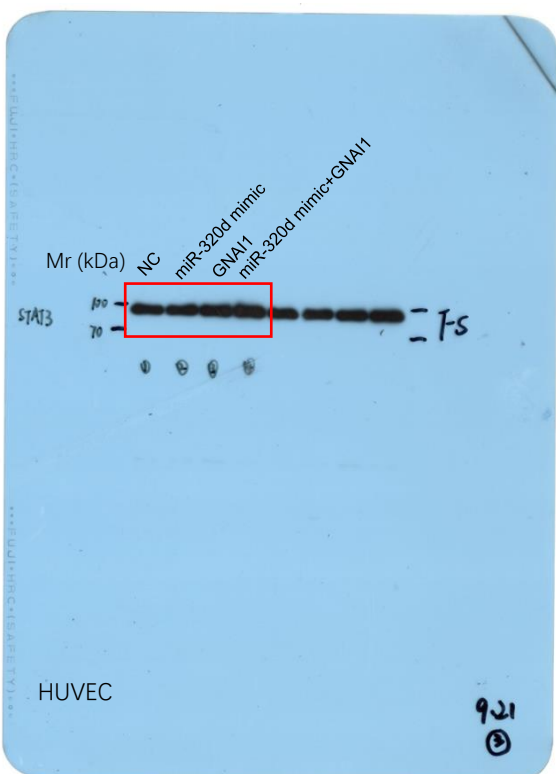

Supplement: Supplementary file 2 — Original western blots [file 41419_2024_7297_MOESM2_ESM.pdf]

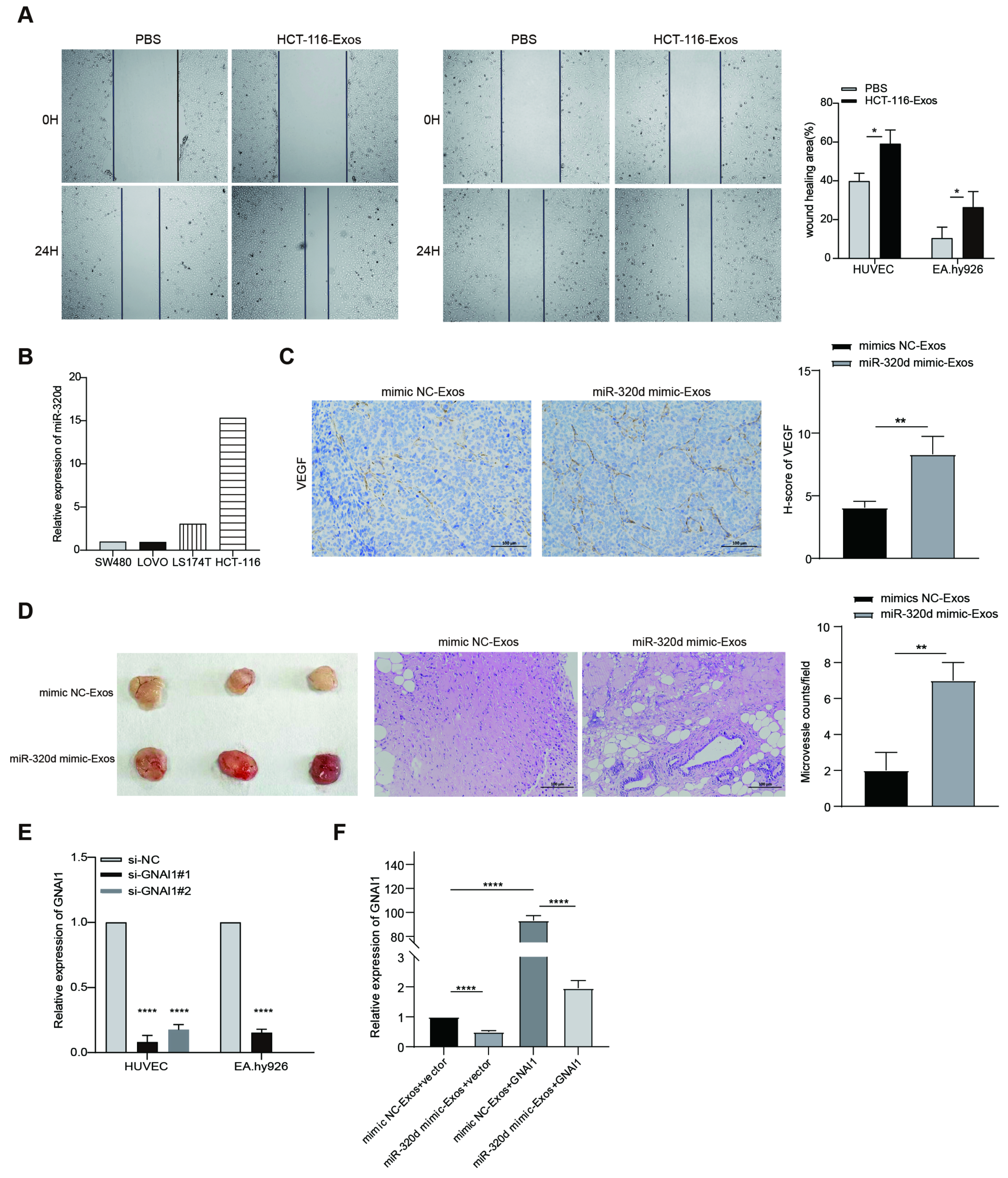

Supplement: Supplementary file 3 — Supplementary FIG 1 [file 41419_2024_7297_MOESM3_ESM.tif]
